# *MPV17* mutation causes neuropathy and leukoencephalopathy with multiple mtDNA deletions in muscle

**DOI:** 10.1016/j.nmd.2012.03.006

**Published:** 2012-07

**Authors:** Emma L. Blakely, Anna Butterworth, Robert D.M. Hadden, Istvan Bodi, Langping He, Robert McFarland, Robert W. Taylor

**Affiliations:** a Mitochondrial Research Group, Institute for Ageing and Health, The Medical School, Newcastle University, Newcastle upon Tyne NE2 4HH, UK; b Department of Neurology, King’s College Hospital, Denmark Hill, London SE5 9RS, UK; c Clinical Neuropathology, Academic Neuroscience Building, King’s College Hospital, Denmark Hill, London SE5 9RS, UK

**Keywords:** Mitochondrial DNA, mtDNA maintenance, Multiple mtDNA deletions, Neuropathy, COX-deficient fibres

## Abstract

Disorders of mitochondrial DNA (mtDNA) maintenance are clinically and genetically heterogeneous, embracing recessive mtDNA depletion syndromes affecting children and adult-onset multiple mtDNA deletion disorders. Here we show that mutation of *MPV17* – a gene implicated in severe, infantile hepatocerebral mtDNA depletion disorders characterised by a loss of mtDNA copies – can also cause clonally-expanded mtDNA deletion and focal cytochrome *c* oxidase (COX) deficiency in skeletal muscle associated with an adult presentation of neuropathy and leukoencephalopathy. The mpv17 protein is therefore intimately involved in both the mtDNA replication and repair processes and associated with both quantitative and qualitative mtDNA abnormalities.

## 1 Introduction

Mitochondrial respiratory chain diseases embrace a clinically and genetically heterogeneous group of inherited human disorders in which neurological involvement is prominent. These can be due to either mitochondrial DNA (mtDNA) mutations, or one of an increasing number of Mendelian disorders in which the primary genetic defect has a secondary consequence on mtDNA stability, resulting in defective nuclear–mitochondrial intergenomic communication [1,2]. This secondary mtDNA defect is expressed in one of two forms: mtDNA *depletion* syndromes are characterised by a quantitative loss of mtDNA copy number, leading to isolated organ or multi-systemic paediatric disease, and have been described in association with recessive mutations in at least nine genes – *POLG*, *PEO1*, *DGUOK*, *TK2*, *RRM2B*, *TYMP*, *MPV17*, *SUCLA2* and *SUCLG1* [3]. With the exception of *MPV17*, all gene products are involved in either mitochondrial DNA replication/repair or the maintenance of mitochondrial deoxyribonucleotide pools; mutations in *MPV17* are associated with hepatocerebral mtDNA depletion but the function of the mpv17 protein remains uncertain [4]. Alternatively, the secondary mtDNA defect can manifest as an accumulation of clonally-expanded mtDNA *deletions* in skeletal muscle, leading to an associated respiratory chain defect which is often demonstrated as a mosaic pattern of cytochrome *c* oxidase (COX)-deficient cells and associated with later onset of clinical symptoms in adult life.

To date, mutations in just four of these genes – *POLG*, *PEO1*, *RRM2B* and recently *TK2* – have been described as causes of both infantile mtDNA depletion syndromes and adult-onset, multiple mtDNA deletion disorders [5–12]. We describe a patient presenting with a severe peripheral neuropathy, leukoencephalopathy and liver involvement due to a homozygous *MPV17* gene mutation leading to multiple mtDNA deletions and focal COX deficiency in skeletal muscle.

## 2 Case report and methods

### 2.1 Case report

The patient is a 21 year-old Pakistani male, born to non-consanguineous parents. He was diagnosed with Hepatitis A (IgM positive) at the age of 3 years and made a full and uneventful recovery. He first developed neurological symptoms at age 14 years, with gradual onset of walking difficulties that progressed over several years to bilateral foot drops requiring orthoses. Over the same period he also developed symmetrical distal numbness and subjective coldness in both feet ascending to the knees, and more mildly in his hands. There was no significant cognitive impairment.

Examination revealed muscle wasting of hands and distal lower limbs. He had clawed toes, but no pes cavus or nerve thickening. He walked with a slow, unsteady gait. Tone was decreased and muscle power was severely weak in a number of distal muscle groups, with no detectable power (MRC grade 0/5) in left abductor pollicis brevis, both first dorsal interossei and both anterior tibialis muscles. While proximal power was normal in the upper limbs, there was symmetrical weakness (MRC grade 4-/5) of hip flexion. All tendon reflexes were absent. Sensory examination was normal in the upper limbs, but revealed decreased perception of pin-prick and light touch distal to the knees, impaired vibration sense to the ankles and absent joint position sense at the toes. There was mild hepatomegaly.

**Fig. 1:**
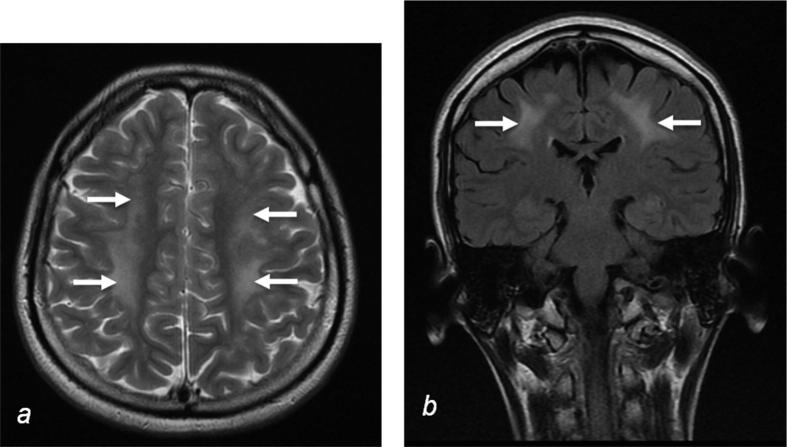
MRI findings. Cranial MRI demonstrates confluent high signal (arrows) in white matter of the frontal and parietal lobes in axial (a, T2 weighted) and coronal (b, FLAIR) sequences, suggestive of leukoencephalopathy.

Liver ultrasound revealed abnormal echogenicity, serum liver enzymes were minimally abnormal (gamma GT 70 U/L, normal range <55 U/L), CT showed heterogeneous liver enhancement, and liver biopsy confirmed patchy steatosis (∼20%) with early bridging fibrosis, but overall clinically there was no significant chronic liver disease. CSF analysis showed an increased protein of 1963 mg/L (normal <500 mg/L) and raised lactate of 4.3 mmol/L (normal 0.7–2.0 mmol/L). Cranial MRI revealed diffuse hyperintense T2 weighted signal in the cerebral white matter (Fig. 1) and cerebellum, but imaging of the brain stem was normal. A previous MRI head scan at age 9 years, performed because of headaches, was normal. Nerve conduction studies confirmed an axonal sensory motor polyneuropathy and sural nerve biopsy showed a severe chronic axonal neuropathy with only occasional surviving myelinated axons.

The patient’s sister developed a progressive, fatal hepatitis at age 9 years. Their father had suffered an episode of hepatitis as a child but made a full recovery. The patient’s mother and older brother are both well and there are no other family members with either liver disease or neuropathy.

### 2.2 Muscle biopsy and mitochondrial DNA analysis

Left quadriceps needle muscle biopsy was performed with informed consent. Histological and histochemical analyses of mitochondrial enzyme activities, including the sequential reaction for COX and succinate dehydrogenase (SDH) activities, were performed using 10 μm serial cross-sections according to standard procedures [13] as was electron microscopy (EM). This study had the relevant institutional ethical approval and complied with the Declaration of Helsinki.

Total DNA was extracted from the muscle biopsy by standard procedures and screened for common mtDNA point mutations. A long-range PCR assay employing a pair of primers (L3965 (nucleotides 3965–3984) and H129 (nucleotides 129–110)) amplified a ∼12.7 kb product in wild-type mtDNA across the major arc of mtDNA to screen for mtDNA rearrangements (GenBank Accession No. NC_012920.1). The level of deleted mtDNA in individual COX-deficient and COX-positive reacting muscle fibres was determined by quantitative real-time PCR of the *MTND1* and *MTND4* regions of the mitochondrial genome as previously described [14]. In addition, the assessment of mtDNA copy number in the patient’s muscle sample was investigated by a real-time PCR assay, which co-amplifies a mtDNA-encoded gene (*MTND1*) and a nuclear housekeeping gene (18S rRNA) [15].

### 2.3 Nuclear genetic analysis

The entire coding regions, including intron–exon boundaries, of the *POLG* (NM_002693), *DGUOK* (NM_080918) and *MPV17* (NM_002437.4) genes were amplified using intronic M13-tailed primers by standard PCR. Purified PCR products (ExoSapIT, GE Healthcare) were sequenced with BigDye Terminator cycle sequencing chemistries on an ABI3130xl Genetic Analyzer (Applied Biosystems) and analysed using Mutation Surveyor software (Softgenetics).

## 3 Results

**Fig. 2:**
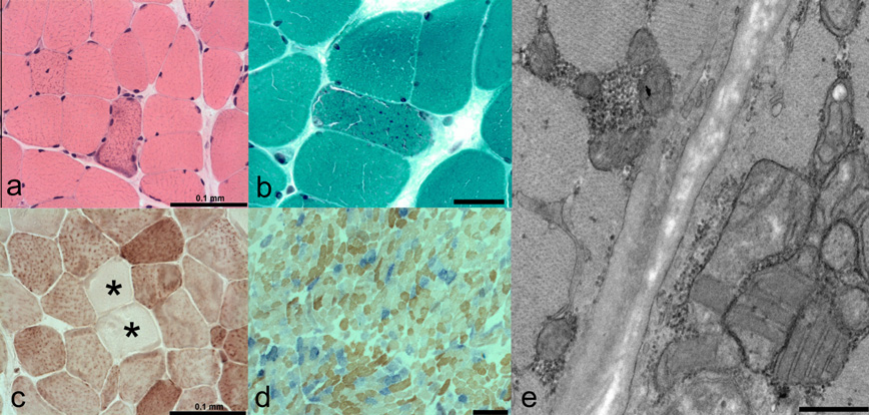
Muscle biopsy findings. (a) H&E, scale bar = 100 μm and (b) Gomori trichrome, scale bar = 50 μm reveal evidence of subsarcolemmal mitochondrial accumulation consistent with “ragged-red” changes in the patient’s biopsy. (c) Several fibres are depleted for COX activity (asterisked; scale bar = 100 μm), which is accentuated following the histochemical demonstration of sequential COX and SDH activities (d; scale bar = 250 μm). (e) Electron microscopy revealed focal subsarcolemmal accumulation of abnormal mitochondria in muscle with typical parking lot-like inclusions, scale bar = 500 nm.

H&E staining of the muscle biopsy was generally unremarkable, revealing only scattered small fibres; there was no evidence of necrosis, phagocytosis, inclusions, fibre splitting or inflammatory infiltrate although occasional fibres displayed internal nucleation (Fig. 2a). Modified Gomori trichrome staining identified accumulation of mitochondria, consistent with a total of four ragged-red fibres representing ∼1% of the total biopsy (Fig. 2b) whilst COX reactivity revealed a number of pale fibres (Fig. 2c). Significant mitochondrial respiratory chain deficiency was confirmed following sequential COX–SDH histochemistry which identified ∼20% COX-deficient fibres throughout the biopsy, implicating mtDNA involvement (Fig. 2d). Electron microscopy demonstrated focal subsarcolemmal accumulation of abnormal mitochondria with typical parking lot-like inclusions in some of the enlarged mitochondria (Fig. 2e).

**Fig. 3:**
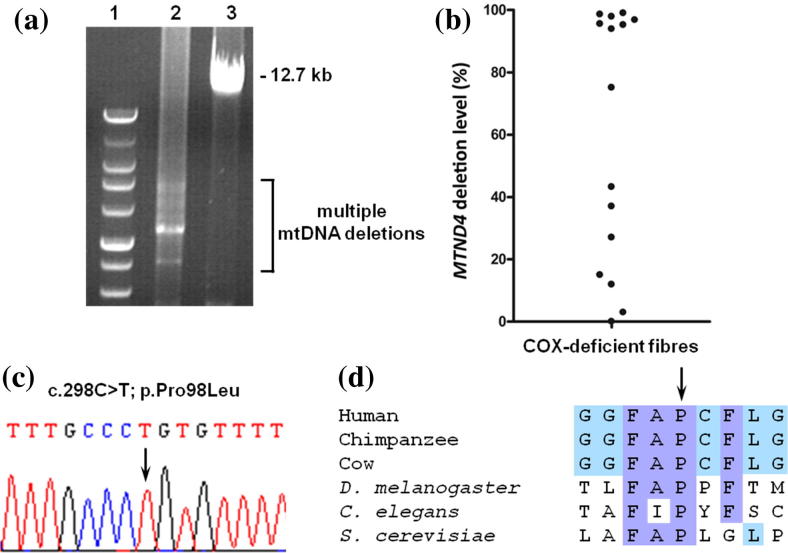
*MPV17* mutation causes multiple mtDNA deletions. (a) Long range PCR of muscle DNA shows significant evidence of multiple mtDNA deletions. Lane 1, DNA size marker; lane 2, patient; lane 3, age-matched control. (b) Quantitative, single fibre real-time-PCR showing many COX-deficient fibres containing high levels of a clonally-expanded mtDNA deletion involving the *MTND4* gene. (c) Sequence chromatogram highlighting the c.298C>T (p.Pro98Leu) *MPV17* mutation identified in our patient. (d) Amino acid alignments of the mpv17 protein from different species confirms a strict conservation of p.Pro98 throughout species from human to yeast.

Real-time PCR demonstrated a normal mtDNA copy number in muscle, whilst common mtDNA point mutations were excluded. Long-range PCR amplification showed the presence of multiple mtDNA deletions (Fig. 3a), confirming a disorder of mtDNA maintenance. Real-time PCR of individual, laser-captured COX-deficient fibres also failed to identify a mtDNA copy number abnormality but clearly revealed that a proportion of fibres had high levels of clonally-expanded mtDNA deletion involving the *MTND4* gene (Fig. 3b), a consistent observation in patients with different nuclear-driven, multiple mtDNA deletion disorders.

Sequencing of three genes (*POLG*, *DGUOK* and *MPV17*) implicated in mtDNA maintenance disorders with hepatocerebral involvement revealed a homozygous missense variant (c.2898C>T; p.Pro98Leu) in *MPV17* which has previously been reported as a recessive mutation associated with mtDNA depletion in liver (Fig. 3c), affecting a highly conserved amino acid within the mpv17 protein (Fig. 3d) [16].

## 4 Discussion

The intra-mitochondrial processes of synthesizing and maintaining mtDNA are dependent not only on a balanced supply of nucleotides, but also on the integrity and concerted action of the mtDNA replication machinery. Disruption of either process can lead to either depletion or deletion of mtDNA, but the factors governing which of these pathological processes prevails are unclear.

Previously, a p.Arg50Gln ‘founder’ mutation in *MPV17* has been associated with Navajo neurohepatopathy (NNH; MIM 256810), a disorder affecting Native American Navajo children, who present with liver disease, failure to thrive, recurrent metabolic acidosis at times of intercurrent infection, corneal scarring, severe sensory and motor nerve impairment and cerebral leukoencephalopathy [17]. Three overlapping phenotypes of NNH have been described with an inverse correlation between age of onset and disease severity. Infantile NNH is an early onset, fatal form of hepatocerebral depletion syndrome while the mildest (classic) form of NNH is characterised by moderate liver dysfunction and progressive demyelination affecting peripheral and central nerve axons. Depletion of mtDNA has been confirmed in liver biopsies [17], but skeletal muscle involvement has not been documented in these patients, or in those patients with other *MPV17* mutations and hepatocerebral depletion syndrome [4,18–20].

The case we present, although phenotypically similar to the classic form of NNH, is unusual in several respects. Importantly, histochemical analysis demonstrated clear evidence of muscle involvement with ragged red fibres and a mosaic pattern of COX deficiency. The latter implicated mtDNA in the pathogenetic mechanism of disease in this patient, but rather than depletion of mtDNA, we identified multiple mtDNA deletions in skeletal muscle, a finding not previously noted in patients with *MPV17* mutations.

Although the parents were non-consanguineous, they were from the same Asian ethnicity and the presence of a homozygous p.Pro98Leu mutation [16] suggests that the parents shared a common ancestor or that this recessive mutation has high population prevalence in this Asian group. Given the experience with NNH, it would seem that a shared common ancestor or founder is most likely; unfortunately, parental samples were not available to confirm carrier status.

The mpv17 protein is an inner mitochondrial membrane comprising four transmembrane domains yet its function and role in the pathogenesis of autosomal recessive disorders of mtDNA maintenance are far from clear in spite of studies in disease models. Deletion of *SYM1*, the yeast orthologue of the *MPV17* gene, causes mitochondrial bioenergetic defects and mtDNA instability [21] whilst a severe mtDNA depletion affecting the liver and CNS tissues was observed in the *MPV17*^−/−^ knockout mouse [22]. Of further interest, the mouse model did exhibit evidence of respiratory chain deficiency in muscle but this was shown to be due to mtDNA depletion. Although our report documents the first patient with *MPV17* mutation leading to multiple mtDNA deletions and associated COX abnormalities in muscle, the clinical and molecular findings in patients with hepatocerebral mtDNA depletion have recently been summarised to investigate possible phenotype–genotype correlations associated with autosomal recessive *MPV17* abnormalities [16]. Mutations are found to be located throughout the mpv17 protein, in both transmembrane and extramembrane domains, but interestingly some may be associated with longer survival and better outcome following liver transplantation; interestingly, these include the p.Arg50Gln NNH mutation and the p.Pro98Leu mutation as observed in our patient, although this has only been described in one case previously [16].

In conclusion, we have identified the presence of clonally-expanded mtDNA deletions in muscle, a tissue not previously recognised to be involved in the pathological process of mpv17-related disease. Although the precise function of this remains undetermined, we confirm this protein as being intimately involved in both the mtDNA replication and repair processes and associated with both quantitative and qualitative mtDNA abnormalities.
